# Pilot feasibility study of a semi-automated three-dimensional scoring system for cervical dystonia

**DOI:** 10.1371/journal.pone.0219758

**Published:** 2019-08-08

**Authors:** Takuto Nakamura, Satoko Sekimoto, Genko Oyama, Yasushi Shimo, Nobutaka Hattori, Hiroyuki Kajimoto

**Affiliations:** 1 Department of Informatics, The University of Electro-communications, Tokyo, Japan; 2 Japan Society for the Promotion of Science, Tokyo, Japan; 3 Department of Neurology, Juntendo University School of Medicine, Tokyo, Japan; Universita degli Studi di Napoli Federico II, ITALY

## Abstract

The objective of this study is to test the feasibility of a semi-automated scoring system for the Toronto Western Spasmodic Torticollis Scale (TWSTRS) severity scale in patients with cervical dystonia. The TWSTRS requires training and experience. We previously developed a system to measure neck angle by analyzing three-dimensional position, obtained using Kinect, a marker-less three-dimensional depth sensor. The system can track patients’ faces and bodies, automatically analyze neck angles, and semi-automatically calculate the TWSTRS severity scale score. We compared the TWSTRS severity scale scores calculated by the system with the video-based scores calculated by a neurologist trained in movement disorders. A correlation coefficient analysis was then conducted. Absolute accuracy was measured using intra-class correlation (ICC) (3,1), with 95% limits of agreement. To analyze the subscales, Cohen's kappa coefficient (κ) was calculated. A p-value of < .05 was considered statistically significant. Thirty patients were enrolled. Their average age was 52.3±16.0 years, and the male to female ratio was 3:2. The average disease duration was 11.3±12.7 years. Total score measurements by the system were significantly correlated with those rated by the movement disorder-trained neurologist (r = .596, p < .05). There was a significant correlation (r = .655, p < .05) with regard to the automated part of the scale. An adequate ICC (3,1) of .562 was obtained for total severity score (p < .001, 95% confidence interval [CI]: .259–.765), while the equivalent score was .617 for the total automated part (p < .001, 95% CI .336–.798). Our three-dimensional motion capture system, which can measure head angles and semi-automatically calculate the TWSTRS severity scale score utilizing a single-depth camera, demonstrated adequate validity and reliability. This low-cost and portable system could be applied by general practitioners treating cervical dystonia to obtain objective measurements.

## Introduction

Cervical dystonia, also referred to as spasmodic torticollis, is a movement disorder that involves abnormal involuntary head posture or movement associated with intermittent or sustained muscle contractions in the neck. Depending on the specific muscles involved, patients may present torticollis (rotation), laterocollis (tilting), antecollis (anterior flexion), retrocollis (posterior extension), or a combination of these, which can lead to debilitating and sometimes painful postures [1]. Treatment options include anticholinergics, muscle relaxants, botulinum toxin injection, and deep brain stimulation [2].

The standardized scale most frequently used to evaluate cervical dystonia is the Toronto Western Spasmodic Torticollis Scale (TWSTRS), which comprises three parts measuring the severity, disability, and pain associated with cervical dystonia[3][4]. The severity scale of the TWSTRS consists of six items: maximal excursion, duration, effect of sensory tricks, shoulder elevation/anterior displacement, range of motion, and time. All of these items are assessed with good inter-rater reliability [5]. However, clinicians require training and experience to evaluate patients using the TWSTRS, because the scale is based on visual observations.

Three-dimensional motion capture systems have been widely used in laboratory settings, but are not usually available in clinical settings because of cost and space limitations [6]. However, marker-less tracking using low-cost, portable motion-sensing devices, such as the Kinect (Microsoft, Seattle, WA), has recently been used to evaluate the biomechanics of movement [7]. The Kinect for Windows v2 (Kinect v2) is a successor model to the Kinect. It is equipped with an RGB-D camera that uses time-of-flight, depth-sensing technology to facilitate a marker-less human pose estimation algorithm [6, 8]. The Kinect v2 has been applied to evaluate gait, balance, and posture, as well as to facilitate rehabilitation [8].

We previously developed a system to measure head and neck angle by analyzing the three-dimensional position obtained by Kinect, and updated to Kinect v2 [9]. The system can track patients’ faces and bodies and automatically analyze the angles of the yaw axis (rotation), roll axis (lateral tilting), and pitch axis (sagittal flexion and extension) of the neck in real time. It can also semi-automatically calculate the TWSTRS severity scale score. The present study aimed to test the feasibility of the semi-automated calculation system in patients with cervical dystonia. We conducted a pilot study to compare the TWSTRS severity score calculated by the semi-automated scoring system with the video-based scores rated by a neurologist trained in movement disorders.

## Methods

### Subjects

The inclusion criteria were as follows: previous diagnosis of cervical dystonia and age between 20 and 80 years. Thirty consecutive patients affected by cervical dystonia of varying severity who had visited the botulinum toxin clinic of the Juntendo University Hospital were recruited. All these patients provided written informed consent for study participation, in accordance with the Declaration of Helsinki, and the study was approved by Juntendo University Hospital Institutional Review Board.

### Kinect v2-based system

We developed a software using the sample code “Face Tracking Basic” in the Kinect for Windows Standard Development Kit (SDK), which detects and tracks human faces captured by Kinect [9]. This program was updated to Kinect v2. In brief, this software can collect real time data based on angles of the yaw axis (rotation), roll axis (lateral tilting), and pitch axis (sagittal flexion and extension) of the neck by tracking the position of the subject’s face, shoulders, and trunk. To conduct this measurement, the system obtains 100 samples in 10 seconds, and we used default smoothing parameters (correction factor: 0.5, smoothing factor: 0.5, jitter radius: 0.05 m, maximum deviation radius: 0.04 m, future prediction: zero frames).

**Fig 1:**
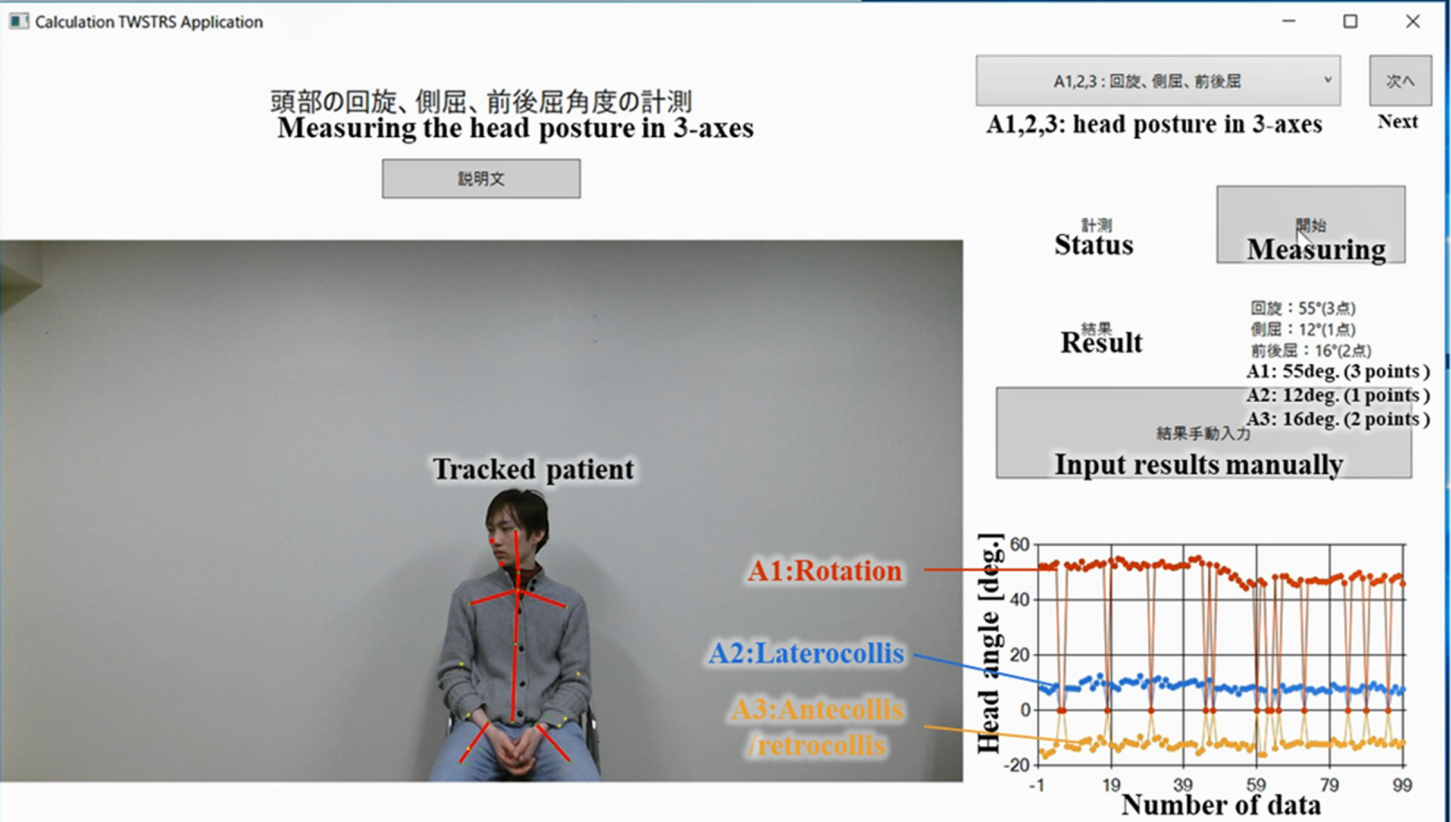
The interface of the system for semi-automated TWSTRS scoring.

To obtain the TWSTRS scores, the semi-automatic measurement program was initiated by clicking the “start” button. Firstly, items A1 (rotation), A2 (laterocollis), and A3 (antecollis/retrocollis), which constitute head angles displacements within three different axes, were automatically calculated based on the measurement conducted during the first 10 seconds (Fig 1). Simultaneously, vertical and horizontal shoulder angles were measured in the background in two axes to allow later scoring. The window was then switched by clicking the “next” button. Items A4 and A5 (lateral and forward/backward shift of the head) were judged by the examiner and manually entered into the system, as the errors of the algorithm when measuring these parameters are not acceptable for clinical use (S1A Fig). The window was then switched to item B (duration factor). The system conducted another measurement for 10 seconds and estimated the percentage of duration of maximal deviation of symptoms (S1B Fig). The window was switched to item C (sensory tricks); the presence or absence of sensory trick was manually entered into the system (S1C Fig). The window was then switched to item D (shoulder elevation) and the examiner instructed the patients to move their left and right shoulders up and down alternatively four to five times. The vertical range of motion of the shoulders was measured until the “end” button was clicked. Subsequently, the examiner instructed the patients to move their left and right shoulders back and forth alternatively four to five times, and the horizontal range of motion of the shoulders was measured until the “end” button was clicked (S1D Fig). The system automatically calculated the score of item D based on the angles and the percentage of durations measured during first 10 seconds, as well as based on the range of the shoulder. The window was then switched to item E (range of motion), and the examiner instructed patients to rotate their head in three axes in the opposite direction to the original position; the system automatically compared the patients’ range of head motion to the central line (S1E Fig). The window was then switched to item F (time for which the patient could keep their neck straight) (S1F Fig); the examiner instructed the patients to keep their head straight and then clicked the “start” button to measure the time for which the patients could maintain their heads within 10° of the neutral position. The measurement automatically stopped after 60 seconds. This was measured twice and the average score was calculated. If the patients could not make their head straight at all, the examiner clicked the “start” button twice to obtain the corresponding score. Finally, by clicking the “finish” button, the items were completed and the scores were recorded in the database. The details of the algorithm for automated scoring of the TWSTRS are described in S1 File and S1 Video shows how the system guides user action. We used the original version of the TWSTRS but range of degrees for classification of laterocollis was modified due to technical reason (See S1 File).

**Fig 2:**
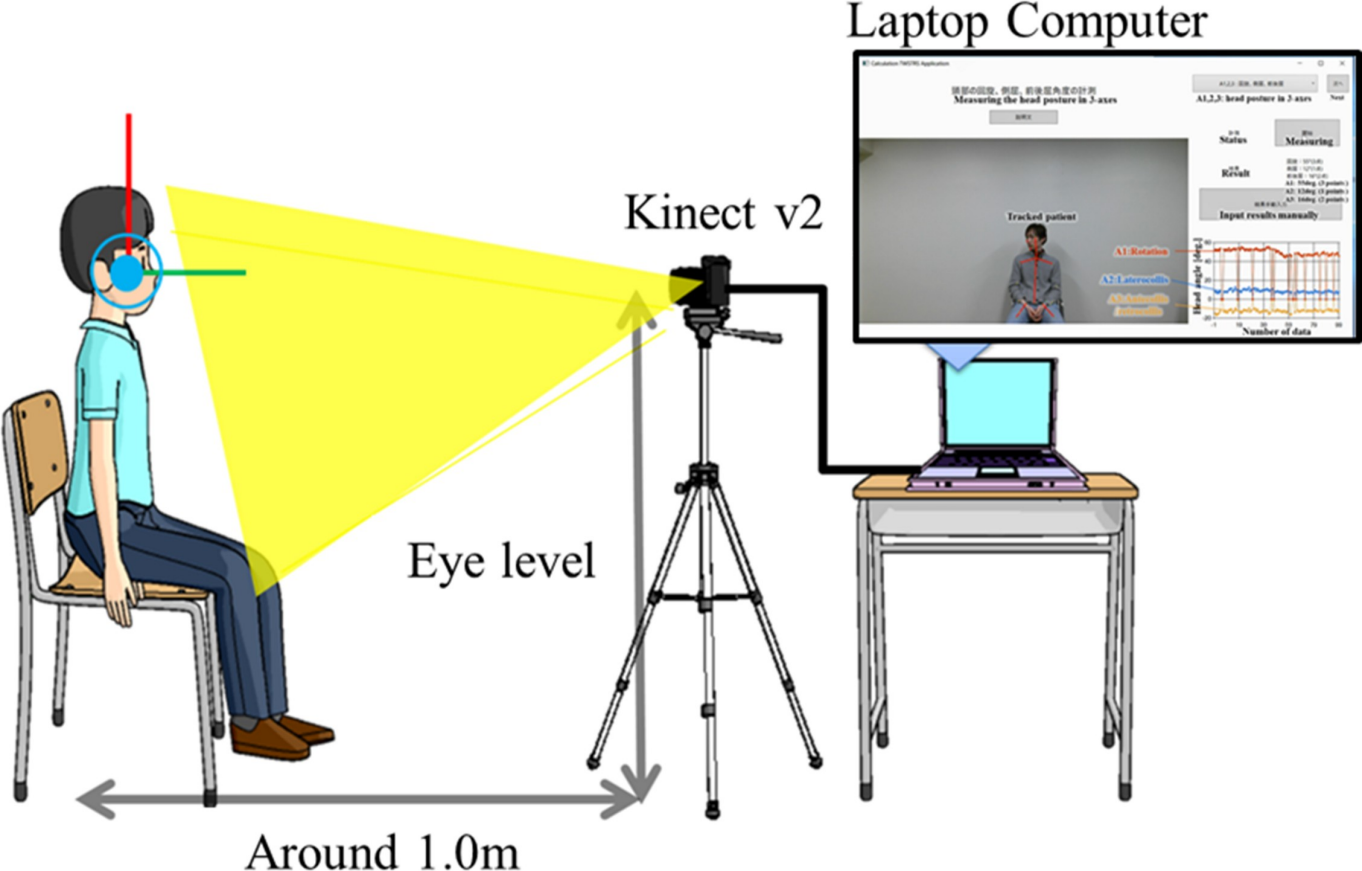
Study equipment.

The Kinect v2 was fixed at the subject’s eye level, and the subjects sat at a distance of 1.0 m away with their knees together in front of a camera that was collimated to the line connecting the articulation points of two knees (Fig 2). Only the frontal body surface was detectable during skeletal tracking.

### Study protocol

An examining neurologist who had been trained using the material of the International Parkinson and Movement Disorder Society (S.S.) performed the TWSTRS severity scale evaluation in all patients under the guidance of semi-automatic TWSTRS scoring software. The whole examination was also videotaped from two directions (front and lateral). Items A1–5 and B were automatically calculated by the system, but items C to F required manual input from the examiner. Using the resulting video, the TWSTRS severity scale was evaluated by a blinded neurologist who had been trained for movement disorders and trained using the material of the International Parkinson and Movement Disorder Society (G.O.).

### Data analysis

Data were analyzed to ascertain the coefficient of correlation between the total TWSTRS score severity scale calculated by the system and the video-based scores obtained by a movement disorder-trained neurologist. Pearson’s r correlation was used to analyze the total score, while Spearman’s correlation was used to assess relative agreement between the two systems. Absolute accuracy was measured using intra-class correlation (ICC [3,2]) and 95% limits of agreement. To analyze the subscales, Cohen's kappa coefficient (κ) was calculated. A p-value of < .05 was considered significant. SPSS statistics 21 was used for statistical analysis.

## Results

In total, 30 patients were enrolled and completed the study. Table 1 shows the characteristics of the participants. Their average age was 52.3±16.0 years, and the male to female ratio was 3:2. The average disease duration was 11.3±12.7 years. This population was considered to represent the general population of patients with cervical dystonia in Japan [10]. All participants were receiving botulinum toxin injections.

**Table 1:** Characteristics of participants.

Patient	Age (years)	Sex	Disease duration (years)	Clinically dominant type of cervical dystonia			
				Antecollis /Retrocollis	Laterocollis	Torticollis	Shift
**1**	56	Female	8	Antecollis	-	Right	-
**2**	80	Male	62		Right	Left	
**3**	40	Male	21			Right	
**4**	43	Female	9		Right	Right	
**5**	35	Male	5			Right	
**6**	77	Male	20			Right	
**7**	33	Female	5	Antecollis	Right	Right	
**8**	58	Male	1	Retrocollis		Right	
**9**	42	Male	6		Left		
**10**	52	Male	1	Antecollis	Left	Left	
**11**	43	Male	9		Right	Right	
**12**	48	Male	0.7			Right	
**13**	28	Male	14				Right
**14**	52	Female	10		Right		
**15**	40	Male	2	Retrocollis		Left	
**16**	72	Male	4		Right		
**17**	79	Male	7		Right	Left	
**18**	67	Male	29	Antecollis	Right	Right	
**19**	54	Male	6		Right	Left	Left
**20**	75	Male	30	Retrocollis		Right	
**21**	65	Female	6	Retrocollis	Right	Left	
**22**	37	Female	4		Right	Right	
**23**	76	Female	16			Left	
**24**	48	Female	10		Right		
**25**	27	Female	1		Right	Left	
**26**	35	Female	7		Right	Left	
**27**	55	Male	27		Right	Right	
**28**	64	Male	0.4		Left	Right	
**29**	51	Female	8		Right	Right	
**30**	37	Female	10	Retrocollis	Left	Right	

**Fig 3:**
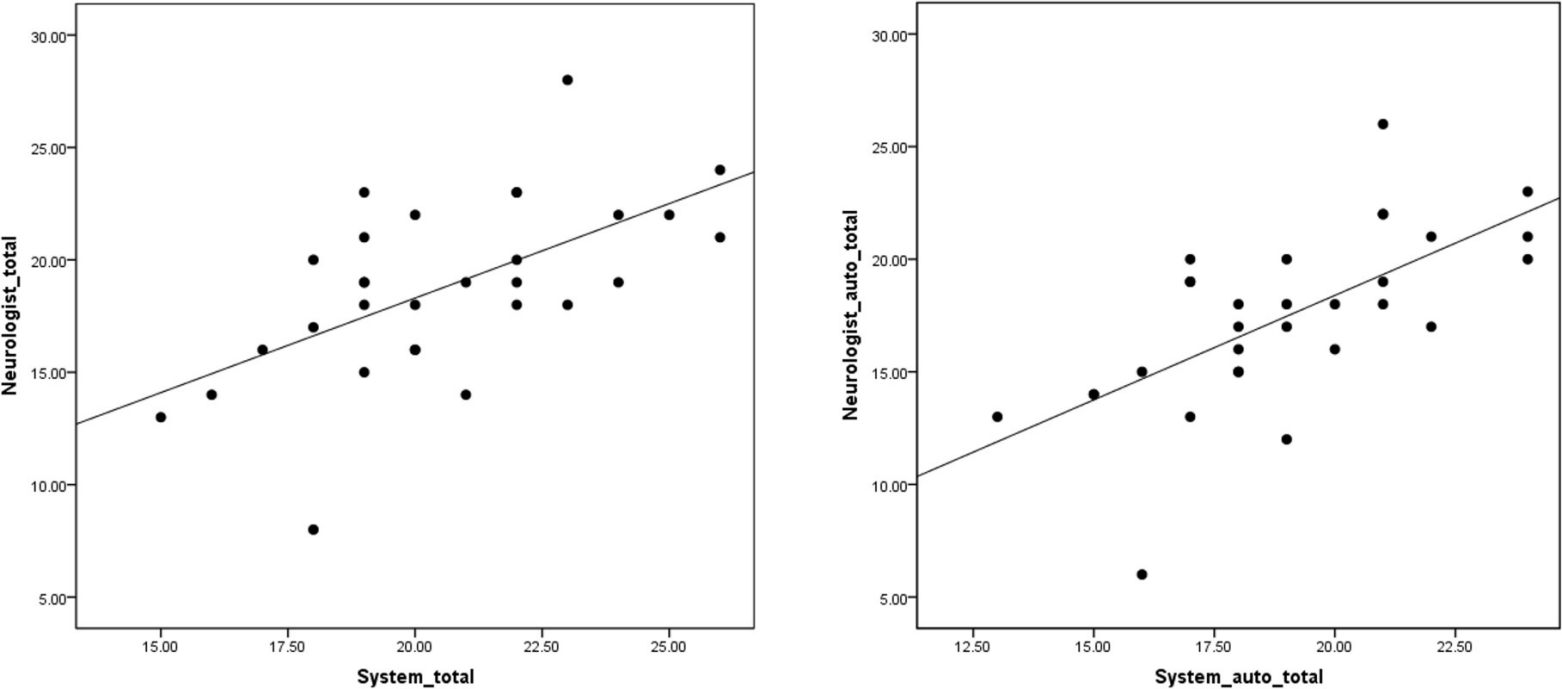
Correlation between a neurologist and the system. Left: Neurologist vs the system (total score), Right: Neurologist vs the system (the automated part without input from the examiner: A1 + A2 + A3 + B + D + E + F).

Table 2 shows the head angles and TWSTRS severity scores calculated by the system, as well as those calculated by the movement disorder-trained neurologist. Measurement by the system was significantly correlated with that by the specialist (r = .596, p < .05; Fig 3, left). With regard to the total score obtained by the automated part without input from the examiner (calculated by adding the subscales automatically analyzed by the system: A1 + A2 + A3 + B + D + E + F), there was a significant correlation between the system and the movement disorder-trained neurologist (r = .655, p < .05; Fig 3, right). Meanwhile, an adequate ICC (3,1) of .562 (p < .001, 95% confidence interval [CI]: .259–765) was obtained in the case of total TWSTRS severity score. Regarding the total automated part, the ICC (3,1) was .617 (p < .001, 95% CI .336–.798). Table 2 shows Spearman’s rho and Cohen's kappa for each subscale. Table 3 shows the scores of the automated parts of the TWSTRS severity scale, as calculated by the system (excluding the parts that required the examiner’s judgment), as well as the equivalent scores obtained by the movement disorder-trained neurologist. Subscale analysis revealed significant correlations between the system and the movement disorder-trained neurologist in A1 (rotation), A2 (laterocollis), A5 (sagittal shift), B (duration factor), C (sensory trick), and F (time). Cohen's kappa coefficient showed significant agreement between the system and the movement disorder-trained neurologist in A1 (rotation), A4 (lateral shift), A5 (sagittal shift), B (duration factor), C (sensory trick), and D (shoulder elevation).

**Table 2:** Raw score of the automated parts of the TWSTRS severity scale calculated by the system vs. score obtained by a neurologist.

Patient	A1 Rotation*			A2 Laterocollis*			A3 Ante/retrocollis**			B Duration		D Shoulder		E Range		F Time	
	RA	S	N	RA	S	N	RA	S	N	S	N	S	N	S	N	S	N
**1**	-49.4	3	3	-19.3	2	1	-10.4	1	1	5	5	3	2	1	1	4	3
**2**	11.7	1	1	-13.1	1	2	-5.3	1	0	4	5	1	1	1	2	4	4
**3**	-1.7	0	0	-15.3	2	2	6.4	1	0	5	5	0	0	1	0	4	4
**4**	-27.0	2	4	-23.4	2	2	-22.6	2	0	4	4	3	1	0	0	4	4
**5**	-59.2	3	4	-14.5	1	0	-14.0	1	0	5	5	2	0	0	0	4	4
**6**	-26.6	2	2	-10.8	1	0	-13.8	1	0	5	5	3	1	1	0	4	4
**7**	-46.3	3	3	-16.3	2	1	-14.7	1	1	5	5	3	1	1	0	4	4
**8**	-44.6	2	4	-16.7	2	1	11.7	1	2	4	3	2	1	1	1	4	3
**9**	11.5	1	1	12.5	1	1	2.7	0	1	5	5	0	2	1	0	4	4
**10**	46.1	3	3	2.3	0	2	-33.5	3	2	5	5	0	2	1	3	4	4
**11**	-6.8	1	0	-8.6	1	1	-22.4	2	0	5	5	1	1	1	0	4	4
**12**	-33.1	2	2	25.0	2	2	-20.2	2	1	4	4	1	1	0	1	4	2
**13**	5.5	1	1	5.8	1	1	-4.5	1	1	4	5	1	1	0	0	3	0
**14**	-5.6	1	1	3.1	1	1	-4.5	1	0	4	5	1	1	1	0	0	0
**15**	31.6	2	2	10.1	1	1	34.4	3	2	5	5	0	1	1	2	4	4
**16**	-32.4	2	2	-21.3	2	2	-29.7	2	1	5	5	3	3	1	1	4	4
**17**	2.9	0	0	-34.9	2	3	-10.1	1	0	5	5	3	2	1	3	4	4
**18**	-33.4	2	3	-25.4	2	2	-13.2	1	1	5	4	2	1	1	3	4	3
**19**	6.6	1	1	3.2	1	0	-20.0	2	0	4	2	0	1	0	0	4	0
**20**	5.5	1	1	-7.1	1	1	-14.4	1	0	4	4	0	1	1	1	4	3
**21**	36.2	2	2	-2.1	0	2	15.3	2	1	4	4	1	1	1	0	4	4
**22**	-17.4	1	2	10.0	1	1	-19.0	2	1	5	5	1	1	0	0	4	3
**23**	16.1	1	1	6.4	1	0	-12.9	1	0	5	5	0	1	1	1	4	2
**24**	-19.3	1	0	-8.1	1	1	-16.1	2	0	5	5	2	1	1	1	0	0
**25**	18.4	1	1	-18.7	2	3	-17.4	2	1	4	5	1	1	1	0	4	4
**26**	13.8	1	1	-11.1	1	2	-8.2	1	1	5	5	0	0	1	0	1	0
**27**	-28.6	2	3	-2.4	0	1	-6.3	1	1	4	4	1	2	1	0	4	4
**28**	-10.6	1	1	4.0	1	1	-11.3	1	1	5	5	0	1	1	0	4	3
**29**	-4.8	1	1	4.5	1	1	-20.5	2	0	5	5	0	0	1	0	4	0
**30**	4.9	1	1	14.4	1	1	15.8	2	1	4	5	1	1	1	1	4	0

RA: raw angle by the system, S: system, N: movement disorder-trained neurologist.

*Negative value represents right.

**Negative value represents anterior.

**Table 3:** Validity and accuracy of the system.

Items	System ***	Neurologist***	Correlation (r/ρ)	ICC/κ
**Total severity scale**	20.6 (2.8)	18.8 (3.9)	.596*	.562*
Total automated scale**	19.0 (2.8)	17.5 (3.9)	.655*	.617*
**A1: rotation**	1.5 (0.8)	1.7 (1.2)	.902*	.624*
**A2: laterocollis**	1.2 (0.6)	1.3 (0.8)	.369*	.227
**A3: antecollis/retrocollis**	1.5 (0.7)	0.7 (0.7)	.181	-.095
**A4: lateral shift**	0.1 (0.3)	0.0 (0.2)	-.50	-.047*
**A5: sagittal shift**	0.0 (0.2)	0.1 (0.3)	.557*	.474*
**B: duration factor**	4.6 (0.5)	4.6 (0.7)	.589*	.444*
**C: sensory trick**	1.5 (0.6)	1.2 (0.6)	.450*	.312*
**D: shoulder elevation**	1.2 (1.1)	1.1 (0.7)	.285	.196*
**E: range of motion**	0.8 (0.4)	0.7 (1.0)	.285	.109
**F: time**	3.6 (1.1)	2.7 (1.6)	.562*	.204

*p < .05

**Calculated by adding the subscales that the system could automatically analyze (A1+A2+A3+B+D+E+F).

***Mean (standard deviation)

## Discussion

We developed a three-dimensional motion capture system that could measure head angles and semi-automatically calculate the TWSTRS severity scale score using only a single-depth camera [9]. The total score and automated part score of the TWSTRS severity scale obtained by the system showed good correlation and ICCs with the equivalent scores obtained by a trained neurologist. Our results are comparable with previously reported ICCs of the TWSTRS; in previous studies, the ICC (2,1) was 0.69–0.75 for neurologists and 0.82 for physiotherapists [5]. Therefore, this semi-automated TWSTRS scoring system provides a low-cost and reliable measurement tool for cervical dystonia in the clinic, and it may help general practitioners or paramedical staff who are unfamiliar with cervical dystonia to evaluate the disease, although the gold standard of three-dimensional motion analysis remains the marker-based optical motion capture system which requires expensive equipment and multiple-depth cameras.

Marker-less motion capture systems still lack accuracy compared to marker-based optical motion capture systems, limiting their usefulness in the clinic [7]. For instance, the tracking accuracy of the Kinect can be influenced by distance from the subject, depth perception, noise, and the subject’s body shape [6], as well as by the limitations of time-of-flight technology [7]. Motion speed can also influence accuracy, because the Kinect v2 only has a capture rate of 30 frames/second [6]. Meanwhile, the low resolution of the Kinect camera reduces smoothness and accuracy [6], and the capture volume depends on the number of cameras [8]. The accuracy can also depend on which body parts are measured [6, 11] and on the axis of evaluation. Indeed, our results showed good correlation between the system and a neurologist with regard to rotation, laterocollis, and sagittal extension/flexion, but poor agreement in the pitch axis of antecollis/retrocollis. This is in line with previous study findings showing that pitch errors were consistently higher than yaw and roll errors when using the Kinect, in contrast to the Vicon-based motion capturing system, perhaps because the Kinect overestimates pitch angle [12]. Indeed, our data showed that the system’s pitch axis score tended to be higher than the neurologist’s score, but this could also mean that the system is more sensitive to changes in pitch angle than the neurologist. Specifically, the system rated the score as “1” if the angle was more than 3º, which might be difficult for a human to detect.

In addition, the key technology used to calculate neck angle in the Kinect v2 SDK system is face detection; extreme angles can disrupt this process and may in turn decrease the accuracy of the system. Allahyari et al. reported moderate to excellent agreement between the Kinect and the electrogoniometer system. In general, angle measurements by the Kinect were good to very good in terms of flexion, extension, lateral flexion, and rotation, but the accuracy was lower when large extension angles were encountered [13]. Darby et al. also reported that large yaw rotations and face disruption produced missing frames, as did combined head and torso rotations greater than 55° in the case of both yaw and pitch rotations [12]. These reports indicated that disruptions in face detection could reduce the accuracy of neck angle calculation.

Despite these concerns of accuracy, one study found that the head pose estimation algorithms of the high-definition face-tracking component of the Kinect v2 SDK could estimate head angle within the range in which the face could be tracked [12], and are comparable with those measured using an electrogoniometer [13]. Indeed, in the present study, none of the subjects’ faces were lost from the sensor’s field of view. In addition, as the root mean square error reported by Allahyari et al. was within 2°, the errors of the Kinect may be acceptable for calculating the TWSTRS scores, which require minimum steps of 15°. Furthermore, recent studies have shown that the accuracy of the single-camera Kinect v2 marker-less algorithm is comparable to that of a marker-based system [8, 11, 14]. In one investigation, the Kinect v2 could accurately measure angular and lateral displacement in most aspects of static postural tests [15]. In addition, Lee et al. showed that the Kinect could accurately measure range of motion in the shoulders [16].

One may argue that cervical dystonia can also be evaluated using two-dimensional video analysis, wearable inertial sensors, and surface electromyography (EMG). However, two-dimensional video assessment may miss sagittal angle information, and wearable devices such as accelerometers must be attached to the patient’s head, which may be uncomfortable. In the objective measurement of movement disorders, surface EMG techniques require specific training, and they are time consuming and sometimes impractical.

In conclusion, our three-dimensional motion capture system, which can measure head angles to calculate the TWSTRS severity scale score semi-automatically using only a single-depth camera, demonstrated adequate validity and reliability. This low-cost, portable system could be used by general practitioners treating cervical dystonia in a clinical setting to obtain objective measurements. The system may also allow precise measurement of the neck angle, so it could lead to the development of a kinematic guide for tailor-made botulinum toxin therapy [17] and home-based rehabilitation of cervical dystonia. In addition, the algorithm could be applied to develop objective measurements in other movement disorders.

## Supporting information

S1 File Algorithm for calculation of TWSTRS scores using the semi-automated scoring system, and information about hardware requirements and cost. (DOCX)

S2 File Minimal data set. (XLSX)

S1 Fig Screenshots of the system in each part of TWSTRS. (TIF)

S1 Video The demonstration video showing how to evaluate the patients using the system. (MP4)
