# Supplementary material for: Pilot feasibility study of a semi-automated three-dimensional scoring system for cervical dystonia
Source: PLoS One. 2019 Aug 8;14(8):e0219758. doi: 10.1371/journal.pone.0219758 (PMC6687132; doi:10.1371/journal.pone.0219758)
Supplement: S1 File — (DOCX) [file pone.0219758.s001.docx]

***Algorithm for calculation of TWSTRS scores using the semi-automated scoring system***

**A. Maximal excursion (calculated based on the measurement during the first 10 seconds)**

**A1. Rotation (turn: right or left)**

The maximal yaw angle measured during the first 10 seconds was applied. If the patient’s torso was not parallel with the Kinect, the rotation angle was adjusted based on the torsion angle of both shoulders (Supplemental Figure 1, left). The angle θ^Y^ was defined as the angle between a straight line connecting the two shoulder points on the horizontal plane (coordinates: X^L^, Z^L^ and X^R^, Z^R^) and a line parallel to the Kinect. The measured face yaw angle was defined as Y. The adjusted yaw “rotation” angle x was defined as Y-θ^Y^. The TWSTRS score was calculated based on the following criteria, considering that the error of the Kinect is within 2°.

Score

0: x < 3°

1: 3 < x < 22°

2: 23 < x < 45°

3: 46 < x < 67°

4: 68 < x < 90°

**A2. Laterocollis (tilt: right or left)**

The maximal roll angle measured during the first 10 seconds was applied. If the subject had shoulder elevation, the tilt angle was adjusted based on the torsion angle of both shoulders (Supplemental Figure 1, middle). The angle θ^R^ was defined as the angle between a straight line connecting the two shoulder points on the axial plane (coordinates: X^L^, Y^L^ and X^R^, Y^R^) and a line parallel to the Kinect. The measured face roll angle was defined as R. The adjusted roll “lateral flexion” angle y was defined as R-θ^R^. The TWSTRS score was calculated based on the following criteria, considering that the error of the Kinect is within 2°.

Score

0: y < 3

1: 3 < y < 15

2: 16 < y≦35

3: y>36

**A3. Antecollis/retrocollis**

The maximal pitch angle measured during the first 10 seconds was applied. If the subject had shoulder elevation, the pitch angle used for score calculation was adjusted based on the torsion angle in the sagittal plane (Supplemental Figure 1, right). The angle θ^P^ was defined as the angle between a line perpendicular to the Kinect and a straight line connecting the coordinates “Spine_shoulder.Z, Spine_shoulder.Y,” and “Spine_MID.Z, Spine_MID.Y”. The measured face pitch angle was defined as P. The adjusted pitch “antecollis/retrocollis” angle z was defined as P-θ^P^. The TWSTRS score was calculated based on the following criteria, considering that the error of the Kinect is within 2°. If z was positive, the patient was deemed to have antecollis, while if z was negative, the patients was deemed to have retrocollis.

Score

0: |z| < 3

1: 3 < |z| < 30

2: 30 < |z| < 45

3: |z| > 45


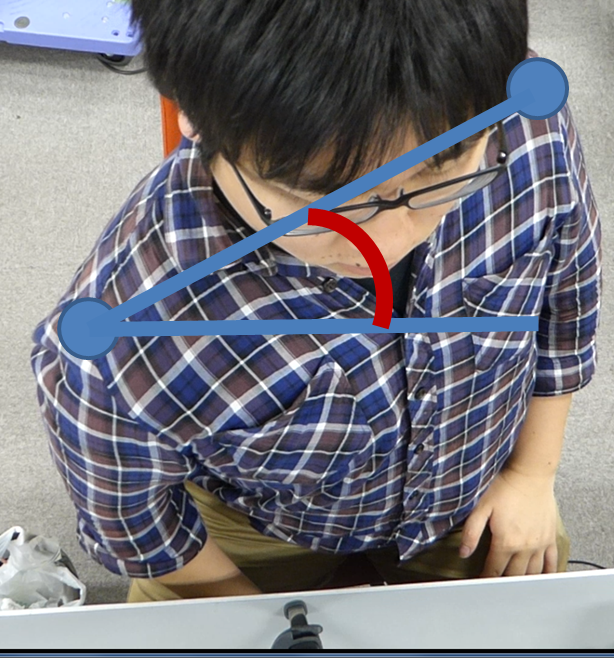

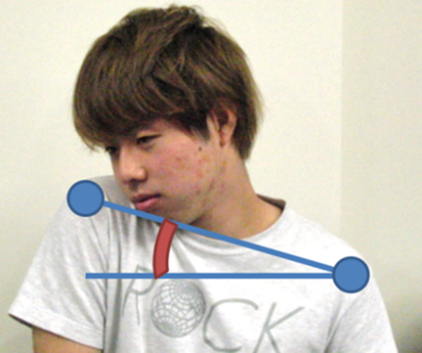

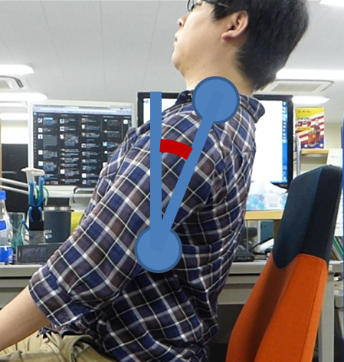


Supplemental Figure 1 (Left: shoulder torsion, middle: shoulder elevation, right: sagittal change in torso)

**A4. Lateral shift**

In the present study, the TWSTRS score for A4 was judged and entered into the system by the examiner.

**A5. Sagittal shift**

In the present study, the TWSTRS score for A5 was judged and entered into the system by the examiner.

**B. Duration factor**

We calculated the duration of minimal excursion (≥ 3° change in any of the axis; T_symptom_) measured during the second 10-second measurement, as well as the ratio between T_symptom_ and the duration of maximal excursion (T_max_) measured during the first 10 seconds. If the affected axis existed more than two axes, the axis showing the largest change in angle was selected for calculation. The score was calculated based on following and subsequently doubled. The term “often” was defined as 2/3.

Score

0: T_symptom_ = 0 sec and T_max_ = 0

1: 0 sec < T_symptom_ < 2.5 sec and T_max_ < T_symptom_*(2/3)

2: 0 sec < T_symptom_ < 2.5 sec and T_max_ > T_symptom_ *(2/3) ,

or 2.5 sec < T_symptom_ < 5.0 sec and T_max_ < T_symptom_*(2/3)

3: 2.5 sec < T_symptom_ < 5.0 sec and T_max_ > T_symptom_ *(2/3),

or 5.0 sec < T_symptom_ < 7.5 sec and T_max_ < T_symptom_*(2/3)

4: 5.0 sec < T_symptom_ < 7.5 sec and T_max_ > T_symptom_*(2/3),

or 7.5 sec < T_symptom_ and T_max_ < T_symptom_*(2/3)

5: 7.5 sec < T_symptom_, T_max_ > T_symptom_*(2/3)

**C. Effect of sensory tricks**

In the present paper, the TWSTRS score for C was judged and entered into the system by the examiner.

**D. Shoulder elevation/anterior displacement**

The vertical and horizontal range of motion of the shoulders were measured (θ^shoulder^) in this item. The ratio of the time for which the patient had shown the symptom (at least 3° ) during the first 10-second measurement was calculated (T_symptomS_). The ratio of shoulder elevation or anterior displacement on the possible range was defined as θ^max^. The θ^max^ was calculated by the following formula;

θ^max^ = MAX ( θ^R^ /θ^shoulder^ and θ^Y^ /θ^shoulder^)

Score

0: θ^max^ < 3°

1: 3° < θ^max^ < θ^shoulder^*1/3

2: θ^shoulder^*1/3 < θ^max^ < θ^shoulder^*2/3 and T_symptomS_ > 7.5 sec,

or θ^max^ > θ^shoulder^*2/3 and T_symptoms_ < 7.5 sec

3: θ^max^ > θ^shoulder^*2/3 and T_symptomS_ > 7.5 sec

**E. Range of motion**

The maximal angle in the opposite direction to that which the patient showed was measured in terms of the range of motion within the three axes; the maximal range of motion in the three axes was used for scoring. We deemed that 30° was the extreme opposite, and that the midline was 0°.

Score

0: Maximal range of motion > 30°

1: 10 < maximal range of motion < 30°

2: 0 < maximal range of motion < 10°

3: Maximal range of motion < 0°,

4: Maximal range of motion - Angle of original position < 5°

**F. Time that the patient is able to maintain the head within 10°of the neutral position**

Time that the patient is able to maintain the head within 10° of the neutral position was measured in this item, and the scores were calculated using the following criteria.

Score

0: Time > 60 sec

1: 46 sec < time < 60 sec

2: 31 sec < time < 45 sec

3: 16 sec < time < 30 sec

4: time < 15 sec, or cannot bring the neck to within 10° of the neutral position.

***Hardware requirements and cost***

The hardware requirements for our system were the same as those previously described for the Kinect v2, which are available on our website (https://docs.microsoft.com/en-us/previous-versions/windows/kinect/dn782036%28v%3dieb.10%29). The minimum system requirements for the Kinect v2 are as follows: OS* Windows 8 (x64) or later version, CPU Intel Corei7 3.1 GHz (or higher), RAM 4 GB (or more), GPU: DirectX 11.0, supported USB: USB 3.0. A PC meeting these specifications was easily obtained for about $1,000, while the Kinect v2 itself costs about $300 and the system only required our developed software. Therefore, the total cost of the entire system requirement is about $1,300.
