# Supplementary figures and images for: Pilot feasibility study of a semi-automated three-dimensional scoring system for cervical dystonia
Source: PLoS One. 2019 Aug 8;14(8):e0219758. doi: 10.1371/journal.pone.0219758 (PMC6687132; doi:10.1371/journal.pone.0219758)

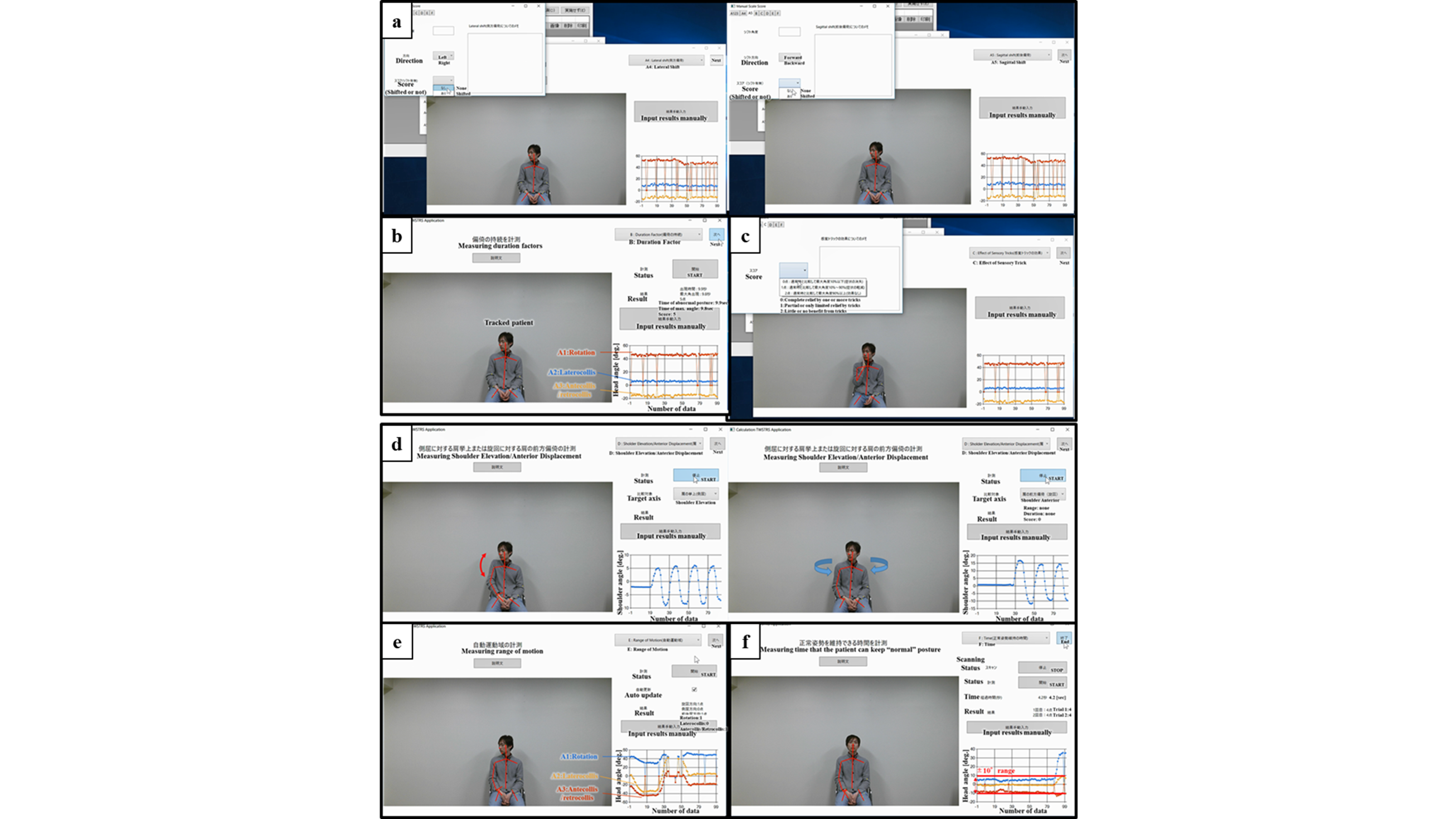

Supplement: S1 Fig — (TIF) [file pone.0219758.s003.tif]
